# Anaerobic metabolism of Foraminifera thriving below the seafloor

**DOI:** 10.1038/s41396-020-0708-1

**Published:** 2020-07-08

**Authors:** William D. Orsi, Raphaël Morard, Aurele Vuillemin, Michael Eitel, Gert Wörheide, Jana Milucka, Michal Kucera

**Affiliations:** 1 Department of Earth and Environmental Sciences Paleontology & Geobiology, Ludwig-Maximilians-Universität München, 80333 Munich, Germany; 2 GeoBio-CenterLMU, Ludwig-Maximilians-Universität München, 80333 Munich, Germany; 3 MARUM—Center for Marine Environmental Sciences, University of Bremen, Bremen, Germany; 4 SNSB—Bayerische Staatssammlung für Paläontologie und Geologie, 80333 Munich, Germany; 5 Department of Biogeochemistry, Max Planck Institute for Marine Microbiology, Bremen, Germany

**Keywords:** Microbial ecology, Microbial biooceanography, Metabolism

## Abstract

Foraminifera are single-celled eukaryotes (protists) of large ecological importance, as well as environmental and paleoenvironmental indicators and biostratigraphic tools. In addition, they are capable of surviving in anoxic marine environments where they represent a major component of the benthic community. However, the cellular adaptations of Foraminifera to the anoxic environment remain poorly constrained. We sampled an oxic-anoxic transition zone in marine sediments from the Namibian shelf, where the genera *Bolivina* and *Stainforthia* dominated the Foraminifera community, and use metatranscriptomics to characterize Foraminifera metabolism across the different geochemical conditions. Relative Foraminifera gene expression in anoxic sediment increased an order of magnitude, which was confirmed in a 10-day incubation experiment where the development of anoxia coincided with a 20–40-fold increase in the relative abundance of Foraminifera protein encoding transcripts, attributed primarily to those involved in protein synthesis, intracellular protein trafficking, and modification of the cytoskeleton. This indicated that many Foraminifera were not only surviving but thriving, under the anoxic conditions. The anaerobic energy metabolism of these active Foraminifera was characterized by fermentation of sugars and amino acids, fumarate reduction, and potentially dissimilatory nitrate reduction. Moreover, the gene expression data indicate that under anoxia Foraminifera use the phosphogen creatine phosphate as an ATP store, allowing reserves of high-energy phosphate pool to be maintained for sudden demands of increased energy during anaerobic metabolism. This was co-expressed alongside genes involved in phagocytosis and clathrin-mediated endocytosis (CME). Foraminifera may use CME to utilize dissolved organic matter as a carbon and energy source, in addition to ingestion of prey cells via phagocytosis. These anaerobic metabolic mechanisms help to explain the ecological success of Foraminifera documented in the fossil record since the Cambrian period more than 500 million years ago.

## Introduction

Foraminifera are one of the most ubiquitous free-living marine eukaryotes on Earth and have been documented in the fossil record since the Cambrian period [1], surviving all mass extinction events involving extensive ocean anoxia [2]. Benthic foraminifera inhabit marine sediments [3], where they can represent up to 50% of the sediment biomass in shallow depths of the seabed [4] and play a significant role in the benthic carbon and nitrogen cycles [5]. Foraminifera are known to be resistant to oxygen depletion and may persist in the benthic community even under the development of anoxic and sulfidic conditions [6–8]. A key to their survival in the absence of oxygen is their ability to perform complete denitrification [9], which appears to be a shared trait among many clades that likely evolved early in the evolutionary history of the group [10]. A better understanding of anaerobic metabolism in Foraminifera under anoxic conditions could illuminate their ecological role in the benthos and explain the ecological success of Foraminifera throughout the Phanerozoic, across multiple mass extinction events, and associated widespread ocean anoxia [2].

To this end, we applied metatranscriptomics to study the active gene expression of anaerobic benthic Foraminifera in anoxic Namibian shelf sediments, and reconstruct their active biochemical pathways. Our transcriptomic analysis showed the anaerobic pathways of ATP production, and revealed the biosynthetic processes that consume ATP. Our data indicate that some Foraminifera affiliated with the genera *Bolivina* and *Stainforthia* are not only surviving under anoxic conditions, but that their transcriptional and cellular activity is stimulated by anoxia. Analysis further shows the anaerobic mechanisms of ATP production which benthic Foraminifera employ to produce sufficient energy to power a multitude of energetically expensive cellular processes in the absence of oxygen. Transcriptional activity of Foraminifera was stimulated by the development of anoxic conditions during a 10-day incubation indicating that many benthic Foraminifera are not only surviving, but similar to a recent study comparing oxygen and nitrate respiration rates in Foraminifera [11], appear to thrive under anoxic conditions.

## Results

**Fig. 1:**
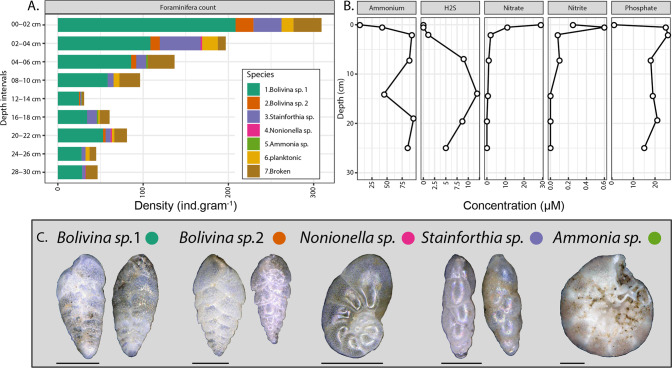
Census count of foraminifera tests and corresponding geochemical profiles in anoxic Namibian sediment. **a** Density of the foraminifera species in the nine intervals processed. Green and brown colors inside the tests indicate the presence of cytoplasm. **b** The changing redox profile of in sediment pore water, note the accumulation of hydrogen sulfide with depth below 6 cm. All O_2_ was below detection immediately below the sediment surface. **c** Representative specimens of the species enumerated; brownish-green color indicates the presence of cytoplasm. Scale bar 100 µm.

Pore water chemical analysis indicated that nitrate and nitrite were consumed quickly at the sediment surface followed by an increased accumulation of ammonium and sulfide with depth (Fig. 1). Intact Foraminifera cells were observed with light microscopy decreased in abundance with increasing depth, but were still present in the deepest part of the core indicating that these Foraminifera cells were living under anoxic conditions (Fig. 1). Most Foraminifera tests observed contained cytoplasm, indicating that they were still alive. Burrowing polychaete worms were observed throughout the core indicating the potential for downward vertical transport of oxidized pore water via bioirrigation processes. However, O_2_ was below detection immediately below (0.5 cm below) the sediment surface. Some polychaete and annelid worms have anaerobic metabolism and the capability to survive in anoxic environments [12] and so the lack of measurable O_2_ indicates that similar to the Foraminifera, some of these burrowing worms may be surviving in the sulfidic sediments through anaerobic metabolism. Throughout the entire core sequence, 95% of the Foraminifera community at all depths was represented by the genera *Bolivina* and *Stainforthia*. Foraminifera absolute abundance had a maximum density at the oxic-anoxic transition at the surface layer of with ~260 benthic foraminifera individuals mostly containing cytoplasm per gram of sediment, followed by a steep decrease until 12–14 cm below seafloor (cmbsf) with 30 individuals per gram of sediment followed by an increase to 80 individuals per gram of sediment at 20–22 cmbsf, coinciding with nitrate-sulfide transition zone (Fig. 1).

**Fig. 2:**
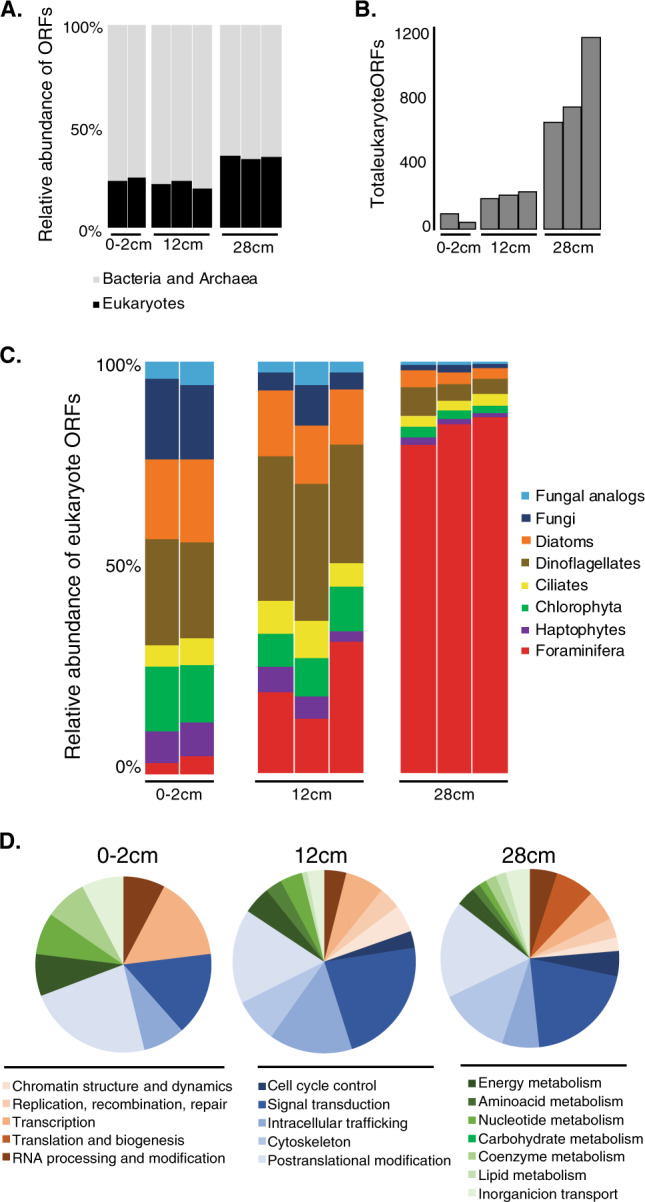
Foraminifera exhibit high levels of gene expression under anoxia. **a** The relative abundance of total expressed ORFs per sample that were assigned to prokaryotes (Bacteria and Archaea) and eukaryotes (including Foraminifera). Multiple histograms per depth represent biological replicates. **b** The total number of ORFs that were assigned to eukaryotes per sample. Multiple histograms per depth represent biological replicates. **c** The relative abundance of expressed ORFs from different protist groups (from **b**), note the dominance of Foraminifera gene expression in the deepest, most anoxic sample at 28 cm. Fungal analogs grouping corresponds to the Labyrinthulomycetes. **d** The relative abundance of functional eukaryotic gene (KOG) families in the three sediment zones that were assigned to expressed Foraminifera ORFs. Pie charts represent average values from the biological replicates shown in **a**–**c**. CT core top sample.

Metatranscriptomes were sequenced to a depth of on average 3.3 (±1.1) million reads per sample (Table S1), excluding one replicate of the core top (0–2 cm) which was sequenced approximately four times deeper (11 million reads). This increased sequencing depth was an attempt to capture additional Foraminifera open reading frames (ORFs) in the metatranscriptomes that went undetected, since the first replicate at 0–2 cm revealed a relatively low relative abundance of Foraminifera ORFs (Fig. 2c). Increasing the sequencing depth to 11 million reads did not result in a proportionally higher abundance of Foraminifera ORFs, indicating that the relatively low fractional abundance of Foraminifera ORFs in the 0–2 cm sample compared with the deeper samples (Fig. 2c) is not a bias of sequencing depth. In the deepest (sulfidic) sample at 28 cmbsf, the relative abundance of Foraminifera expressed ORFs was far greater than all other groups of protists identified in the transcriptomes, reaching >80% of total eukaryotic ORFs and this was consistent across all three replicate metatranscriptomes (Fig. 2c). The relative level of gene expression by the Foraminifera increased with depth, because the total number of unique expressed protein encoding ORFs assigned to Eukaryotes increased (Fig. 2b) which could be attributed to a greater relative abundance of Foraminifera ORFs in the deeper samples (Fig. 2c). A higher number of unique ORFs expressed by Foraminifera cannot be explained by a reduction in gene expression from other groups. Clearly, some of the Foraminifera that were observed with intact cytoplasm in the deeper part of the core (Fig. 1) increase their gene expression under anoxic conditions (Fig. 2b, c).

**Fig. 3:**
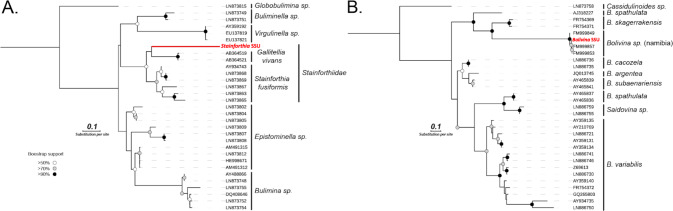
Phylogenetic analysis of Foraminifera affiliated 18S rRNA sequences recovered from the metatranscriptomes. Two 18S rRNA sequences were detected in the metatranscriptomes that are affiliated with the (**a**) Stainforthiidae family and **(b)** *Bolivina* genus. The sequence affiliated to the Stainforthiidae family clearly cluster with the only two representative genera of the family, *Stainforthia* and *Gallietellia* but the position of the metatranscriptomic 18S rDNA sequence is not clearly resolved, but intact test of *Stainforthia* were observed in the sample (See Fig. 1). The metatranscriptomic 18S rDNA sequence related to *Bolivina* is nearly identical to reference sequences deposited on NCBI and that were generated from *Bolivina* specimens collected in Namibia in previous studies. Furthermore, *Bolivina* specimens dominated the morphological assemblages within the core (Fig. 1). The *Bolivina* and *Stainforthia* 18S rDNA contigs were generated by semiautomated greedy extension of 18S rDNA OTUs with trimmed metatranscriptomic paired-end reads (see “Methods”).

Phylogenetic analyses of two Foraminifera 18S rRNA sequences recovered from the metatranscriptomes had closest affiliation to previously reported *Stainforthia* and *Bolivina* 18S rRNA sequences, also recovered from anoxic Namibian sediments (Fig. 3). Some metatranscriptome studies use an rRNA depletion step to reduce the amount of rRNA sequences in order to capture a greater percentage of mRNA sequences, which could potentially bias the recovered rRNA sequences. However, we did not use an rRNA depletion in preparing our metatranscriptome libraries, so any associated detection biases of rRNA sequences due to depletion methods should not have affected our rRNA data. *Stainforthia* and *Bolivina* tests containing cytoplasm were observed in the core, their relative abundance gradually increased with depth, and *Bolivina* was the most abundant genus observed (Fig. 1). Successful detection of its expressed 18S rRNA confirms that our metatranscriptomic approach captured the activity of this numerically dominant group. This is also was reflected in the read mapping statistics (Fig. S1), which support the ratios observed based on counts of cytoplasm-containing tests. Namely, the *Bolivina* sp. 18S rRNA fragment had an average coverage of 125×, whereas the 18S rRNA from the comparatively less abundant cytoplasm-containing tests from *Stainforthia* sp. (Fig. 1) had a lower mean coverage of 34× (Fig S1).

In contrast to 18S rRNA sequences, metatranscriptomic ORFs had the highest similarity to previously sequenced genomes and transcriptomes of *Ammonia*, *Elphidium*, *Rosalina*, and *Globobulimina* cells (Fig. S2), the very few previously sequenced transcriptomes derived from Foraminifera [10, 13, 14]. We could not find publicly available genome or transcriptome data from *Stainforthia* or *Bolivina* to include in our database for annotating the metatranscriptome data. Thus given that we could only detect 18S rRNA from *Stainforthia* and *Bolivina* (Fig. 3) in the metatranscriptomes (and none from *Ammonia*, *Elphidium*, *Rosalina*, and *Globobulimina)*, we assume that most of the ORFs with highest similarity to Foraminifera are likely derived from the numerically dominant *Stainforthia* and *Bolivina* cells observed in the core (Fig. 1), but have top hits to other Foraminifera (e.g., *Ammonia*, *Elphidium*, *Rosalina*, and *Globobulimina*: Fig. S2) since *Stainforthia* and *Bolivina* transcriptomes are missing in our database. We then proceeded to analyze these Foraminifera-derived ORFs in the metatranscriptomes to gain insights into possibly anaerobic biochemical pathways and physiologies, after annotating all of the Foraminifera-derived ORFs against the clusters of Eukaryotic Orthologous Genes (KOGs) database [15].

**Fig. 4:**
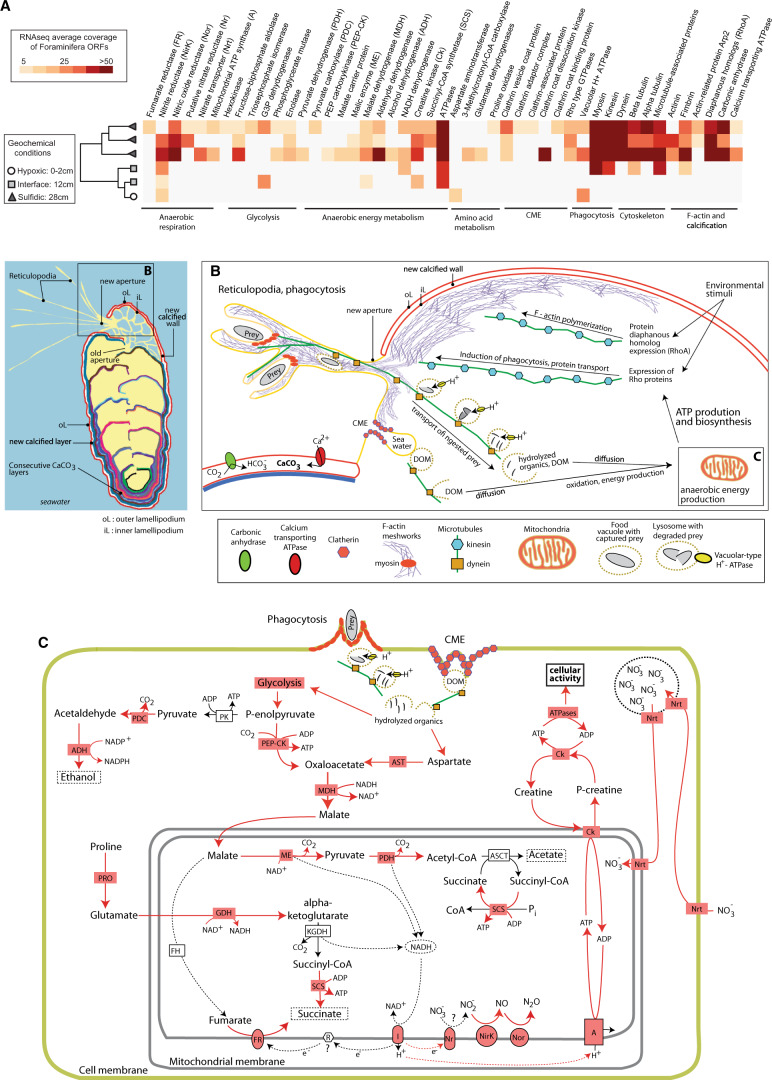
Expression of Foraminifera ORFs involved in key anaerobic physiologies. **a** Heatmap displaying the expression levels of Foraminifera ORFs involved in anaerobic energy production and physiology. Dendrogram shows hierarchical clustering (UPGMA) of the samples based on the RNA-seq data. One metatranscriptome from the core top and one from the 12 cm sample did not have any detectable expression of the ORFs of interest and are thus not shown. **b** Reconstruction of anaerobic cellular activities in Foraminifera including biomineralization, phagocytosis, CME, and transport of ingested cargo (Banning, Novel strains isolated from a coastal aquifer suggest a predatory role for flavobacteria) based on the gene expression data shown in **a**. **c** Reconstruction of potential anaerobic energy production pathways in Foraminifera based on the gene expression data shown in **a**. Red colors show genes that were expressed, red arrows show reactions that are predicted to occur based on the expression of the corresponding gene. Where expressed, gene abbreviations (e.g., Nrt) are shown in red boxes, that correspond to the same labels in **a**. Gene abbreviations displayed with white background are present in the genome of the benthic foraminifera species *Globobulimina turgida* and *G. auriculata* [10], but expression was not detected. These include FH fumarase, KGDH alpha-ketoglutarate dehydrogenase, PK pyruvate kinase, and ASCT acetate:succinate CoA-transferase. This updated representation of Foraminifera anaerobic energy production is modified from anaerobic energy metabolism pathways in eukaryotes that were previously reviewed [39, 40].

Expression of foraminiferal KOGs showed that at all depths the transcriptional activity was dominated by genes involved in cell cycle and cell signaling processes, namely cell cycle control, signal transduction, intracellular trafficking, cytoskeleton, and posttranslational modification (Fig. 2d). The expression of genes involved in translation and biogenesis was detected only in the deepest, anoxic sample, further indicating increased cellular activity (e.g., protein synthesis) under the anoxic conditions. There was also a general trend of decreasing energy production and conversion (KOG category C) with depth, together with an increasing expression of genes involved in signal transduction under anoxic conditions (Fig. 2). The vertical geochemical pore water profiles in the sediment core (Fig. 1) show that the samples can be grouped in two categories: (1) depths at which nitrate is present (core top and 12 cmbsf), and (1) depths were no nitrate is present (28 cmbsf). Comparing the gene expression data from Foraminifera in samples assigned to these two groupings had a statistically significantly difference (Fig. 4a: analysis of similarity [ANOSIM]: *P* < 0.01) in expression of Foraminifera ORFs based on pore water chemistry and associated redox state (e.g., in the presence or absence of pore water nitrate).

The Foraminifera gene expression data indicate three possible anaerobic mechanisms of ATP production in benthic Foraminifera: (1) substrate level phosphorylation of sugars and amino acids via glycolysis and fermentation, (2) use of fumarate as a terminal electron acceptor via fumarate-NADH reductase, and (3) dissimilatory reduction of nitrite to generate proton gradient at the membrane for generation of ATP via ATP synthase (Fig. 4c). Moreover, the data indicate that dephosphorylation of the phosphogen creatine phosphate allows for reserves of the high-energy phosphate pool to be maintained for sudden demands of increased energy under anaerobic conditions, for example phagocytosis which involves bursts of energetically demanding activity. A partial foraminiferal denitrification pathway [10] was expressed including a putative dissimilatory nitrate reductase (Nr), dissimilatory nitrite reductase (NirK), and nitric oxide reductase (Nor) (Fig. 4a). In addition, genes encoding foraminiferal nitrate transporters [10] were expressed indicating active transmembrane nitrate transport. No ORFs with significant similarity to NarK type nitrate/nitrite antiporters, that are common in denitrifying bacteria [16], were detected in the Foraminifera transcriptomes. Apparently, these anaerobic energy production mechanisms produce sufficient ATP in the Foraminifera cells to fuel energetically costly biosynthesis pathways including modification of the cytoskeleton and clathrin-mediated endocytosis (CME) (Fig. 4).

The anaerobic energy production mechanisms were also apparently able produce sufficient ATP in the Foraminifera cells to fuel biomineralization, consistent with prior experimental evidence that Foraminifera can calcify under anoxia [17]. Of note are the expression of Foraminifera ORFs encoding F-actin proteins, that have been shown experimentally to be involved in the biomineralization of the calcium carbonate test [18]. Foraminiferal genes encoding ORFs with similarity to protein diaphanous homolog 1 [19] were also expressed (Fig. 4a), which respond to environmental stimuli and are responsible for actin nucleation and elongation factor required for the assembly of F-actin structures [19]. Since F-actin is required for biomineralization and calcification of the Foraminifera test [18], we speculate that expression of DIAPH1 was involved in the ongoing calcification of Foraminifera under the anoxic conditions.

Foraminiferal genes encoding Rho proteins were expressed, that are responsible for the induction of phagocytosis [20, 21]. Furthermore, Foraminiferal vacuolar-type H+ ATPases were expressed, which are responsible for lysing digested prey cells inside food vacuoles after phagocytosis [12]. No Rho proteins or vacuolar-type H+ ATPases were found to be encoded in contigs from non-Foraminifera eukaryotic groups in the same samples. Instead, all expressed ORFs that were annotated with significant similarity to Rho proteins and vacuolar-type H+ ATPases in the KOG database had highest similarity to Foraminifera genomes and transcriptomes in our database. While it is difficult to assess phagocytosis from homology based transcriptome analysis alone, based on expression of these genes from Foraminifera known to be active predators that phagocytose prey in anoxic habitats [1], and the lack of the expression of these genes from other microbial eukaryote groups known to prefer oxygenated planktonic habitats (e.g., Chlorophyta, dinoflagellates, and haptophytes: Fig. 2c), we speculate that these results are an indication that the Foraminifera were using the encoded Rho proteins and vacuolar-type H+ ATPases proteins to perform phagocytosis under anoxic conditions. Foraminifera ORFs were also expressed that encoded tubulins, kinesin, and dynein, the latter two which are responsible for sending and receiving cellular cargo to and from the membrane, respectively (Fig. 4). The expression of ORFs encoding “unconventional” myosin I, II, and VII [22] from Foraminifera further indicate active phagocytosis because these are nonmuscle myosins that accumulate at the “phagocytic synapse” (Fig. 4b), the point of contact between the pseudopodia and prey cell. This suggests a role for contractile motors proteins during particle internalization [23]. Pseudopod extension and engulfment has been shown experimentally to be mediated by myosin II that is recruited to the phagocytic synapse [24]. However, in addition to phagocytosis, myosin motor proteins play an important part in several cytoskeletal processes involving movement such as cell adhesion, cell migration, and cell division [22]. Thus, it is likely that myosins expressed by the Foraminifera under anoxic conditions play a role in a wide range of cellular processes that require force and translocation, for example their motility through the sediment matrix as they search for prey. Clathrin-encoding genes from Foraminifera were also expressed in two samples (at 28 cmbsf) that are involved in CME, an additional form of endocytosis and involves an invagination of the membrane via clathrin proteins [25]. CME results in much smaller vesicles (30–200 nm) compared with those obtained from phagocytosis (500–9000 nm) [25] and are used to ingest signaling molecules and other forms of dissolved organic matter.

**Fig. 5:**
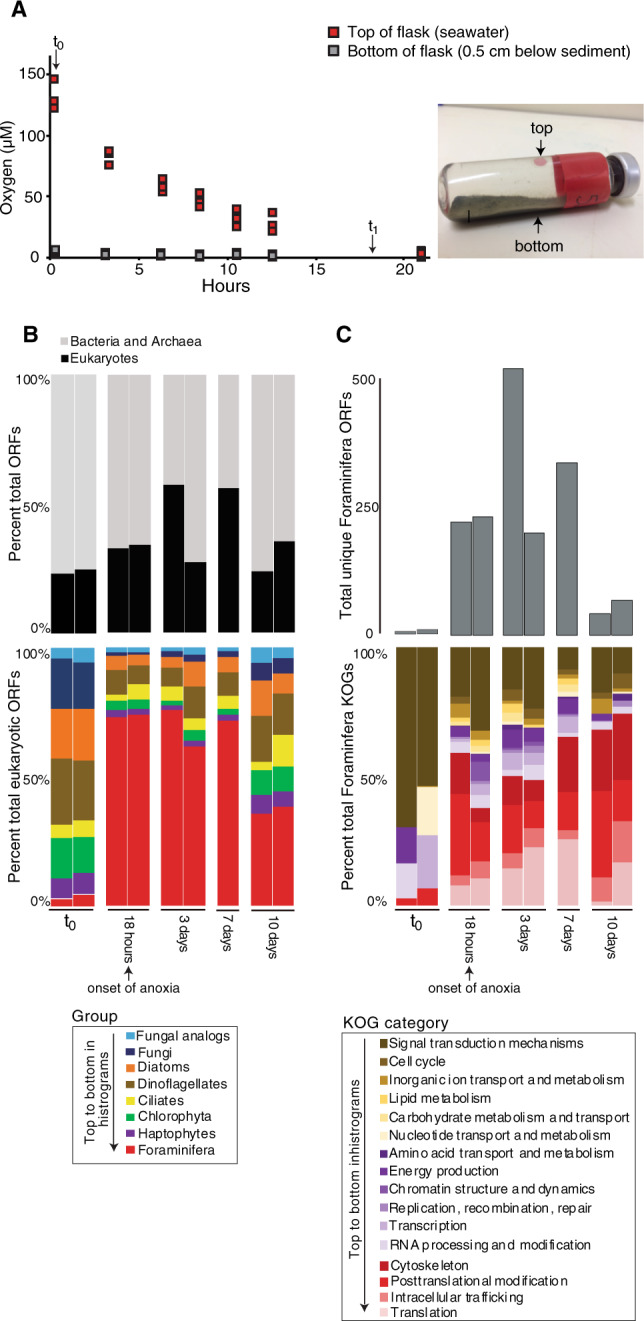
Oxygen consumption and Foraminifera gene expression in a 10-day incubation. **a** Oxygen consumption at the top (in seawater) and bottom (underneath the sediment) of the incubated sediments, the photo shows the experimental setup and the positioning of the two oxygen sensor spots where measurements were made. After the onset of anoxia after 20 h, the top and bottom of the flask remained anoxic for the duration of the incubation. The flask was incubated in the dark at 10 °C. The replicate measurements at each time point are those made on the four separate flasks incubated for the *t*_1_, *t*_2_, *t*_3_, *t*_4_ timepoints. **b** The relative abundance of bacterial and archaeal ORFs compared with total eukaryotic ORFs (top), and the relative abundance of ORFs from eukaryotic groups detected in the metatranscriptomes (bottom). **c** The number of ORFs assigned to Foraminifera (top) and the relative abundance of KOG categories within those foraminiferal ORFs (bottom).

A 10-day incubation of sediment collected from the oxygenated core top layer (0–2 cm), showed that benthic Foraminifera increased their gene expression 20–40-fold after the development of anoxic conditions (Fig. 5a, b). This dramatic increase in gene expression was observed after oxygen consumption declined over the first 20 h of the incubation, which was consistent between all biological replicates (Fig. 5a, b). After the development of anoxic conditions, the relative abundance of Foraminifera gene expression decreased after 10 days but still remained five to ten times higher than the *t*_0_ values (Fig. 5b, c). With the onset of anoxic conditions, the Foraminifera expressed higher numbers of genes involved in the cytoskeleton, translation, posttranslational modification, and intracellular trafficking, which increased progressively with time (Fig. 5c). Differences in the expression of Foraminifera KOGs (*n* = 536 unique KOGs) were found to be statistically significant (ANOSIM: *R*^2^ = 0.75, *P* = 0.001) between the following sample groupings in the incubation according to different stages of oxic and anoxic conditions: (1) oxic conditions at *t*_0_ (*n* = 2 samples), (2) initial onset of anoxia between 18 h and 3 days (*n* = 4 samples), (3) prolonged anoxia from 7 to 10 days (*n* = 3 samples).

## Discussion

On the Namibian continental shelf, Foraminifera live below the seafloor down to ca. 28 cmbsf in an anoxic environment that is extremely high in sulfide [26]. The dominance of *Bolivina* throughout the core and our detection of their 18S rRNA, even into the anoxic depths, is consistent with the known affinity of *Bolivina* for oxygen-depleted habitats [27], including the studied region as it was observed previously in sulfidic sediments at multiple coring locations on the Namibian shelf [26]. The “trophic oxygen model” predicts that the dynamic nature of microhabitats allows Foraminifera to migrate up and down in the sediment with the prevailing redox conditions [28]. Hence, since we sampled during the southern Winter when bottom water oxygen levels in the Namibian OMZ are higher [29, 30], it is possible that the penetration depth of the Foraminifera extended relatively deep because of the higher oxygen concentration at the sediment surface.

Although the diversity of Foraminifera is well constrained by morphological studies, the group is not yet well represented in transcriptomic and genomic databases. The recently large transcriptome sequencing effort of microbial eukaryotes helped to alleviate this problem [14], since it included several Foraminifera that we could add to our database. Nevertheless, because of the relatively low number of sequenced genomes and transcriptomes from Foraminifera (compared with bacteria for example), our metatranscriptome approach cannot distinguish between ORFs derived from different Foraminifera species. The ORFs assigned to Foraminifera here thus serves as a “group averaging”, but should correspond to genetically similar populations since the de novo assemblies that are used to build the contigs from the RNA-seq data are based on genetic similarity (see “Methods”). Furthermore, our metatranscriptomes contained the complete 18S rRNA sequence (Fig. 3) from the most abundant taxa, i.e., *Bolivina* sp. and *Stainforthia* sp. (Figs. 1, 3, and S2) and thus we are confident that the ORFs assigned as Foraminifera are derived primarily from these cytoplasm-containing Foraminifera tests that we could enumerate in the core (Fig. 1). Despite the presence of two morphological different *Bolivina* species in the core, we could not find signs for the active expression of the 18S rRNA in the second species. This indicates that most of the identified foraminiferan metatranscriptomic expression likely comes from one of the *Bolivina* species in addition to *Stainforthia* sp.

The composition of the community is important when using relative abundances for normalization to compare between samples, since the comparison can be biased if certain groups are present at high abundance in some samples, and missing in others. In our sampled core however, all the same major protist groups were present at all depths, albeit in different proportions (Fig. 2c), indicating that differences in relative abundance of groups can be compared between depths. Thus, the differences in relative levels of gene expression of Foraminifera between depths cannot be explained solely by a different community composition at those depths. Similarly, in this same core the composition of the bacterial and archaeal community also does not change drastically with sediment depth [31]. A higher number of unique ORFs would be expected to increase with a higher proportion of Foraminifera as this is associated with increased sequencing depth leading to the recovery of more of the less expressed ORFs. However, the concentration of Foraminifera cells is six times lower in the deepest anoxic samples (Fig. 1), but at this depth the Foraminifera have eight- to tenfold higher relative levels of gene expression compared with those at the surface (Fig. 2). An eight- to tenfold increase in gene expression, from a community with a sixfold lower concentration of cytoplasm-containing cells, is a strong indication for increased transcriptional activity per Foraminifera cell. There was a relatively higher percentage of Foraminifera transcripts involved in energy production (KOG:C) in the surface sample, compared with the deeper anoxic samples (Fig. 2d). We speculate that this could be due to the availability of O_2_ at the sediment seawater interface, which can fuel aerobic respiration and increased energy metabolism.

Foraminifera are predators, and are thought to act primarily as heterotrophs utilizing ingested prey cells as carbon sources for growth [32]. Our gene expression analysis provides insights into the possible mechanisms of prey acquisition, and the metabolic processing of the ingested material. The expression of ORFs encoding Rho proteins by Foraminifera indicate an active induction of phagocytosis, since Rho proteins function in actin dynamics during phagocytosis [20, 21]. Myosin motor proteins are recruited to the cell membrane during phagocytosis in order to envelope and capture prey particles [33], and the prey then enter the phagocytosing cell as a food vacuole [25]. Food vacuoles are then transported into the cell via dynein along microtubules, where the contents are digested under acidic conditions via the activity of vacuolar-type H^+^ ATPases [25] (Fig. 4c). Such proton pumping ATPases are responsible for lysing digested prey cells inside food vacuoles after phagocytosis, where the acidified lysosomal vesicles are loaded with digestive enzymes [25]. The metatranscriptome data indicate that under anoxic conditions, the Foraminifera metabolize the hydrolyzed organics for ATP production via fermentation and fumarate reduction, and dissimilatory nitrite reduction (Fig. 4c). Because cells are mostly protein, anaerobic fermentation of ingested prey cells by Foraminifera may include amino acid fermentations. By weight, exponentially growing cells are made of roughly 50–60% protein, 20% RNA, 10% lipids, 3% DNA, 10–20% sugars as cell wall constituents, and some metabolites [34]. Amino acid fermentations provide roughly one net ATP per amino acid fermented [25].

The fumarate reduction during anaerobic energy metabolism in eukaryotes is usually associated with rhodoquinone (RQ) as an electron carrier, and RQ generally replaces ubiquinone as an electron carrier in the electron transport chain after the switch from aerobic metabolism to anaerobic metabolism [35]. The switch to RQ synthesis during anaerobic metabolism is controlled by the polyprenyltransferase COQ-2 [35], but we could not find any expressed ORFs in our metatranscriptomes with significant similarity to this gene. Future controlled experiments involving the switch from anaerobic conditions could test whether Foraminifera indeed use RQ as an electron carrier during anaerobic energy metabolism.

In addition to hydrolyzed organics from ingested prey, the transcriptomes suggest that CME is another mechanism by which Foraminifera could utilize both high- and low-molecular weight dissolved organic matter (dissolved in the pore water of the sediments) under anoxic conditions. Experiments using ^13^C-labeled diatom prey showed that under anoxic conditions the benthic foram *Ammonia tepida* reduced the number of phagocytosed diatom cells, and the ingested cells were apparently not digested inside vacuoles but remained intact after 4 weeks [36]. If a decreased utilization of ingested prey for energy production is a general feature of anaerobic Foraminifera, it is possible that organic matter obtained via CME (Fig. 4b, c) becomes a relatively more important carbon source as opposed to ingested prey cells.

Eukaryotic fermentations can produce a variety of end products, and our data indicate the possibility for Foraminifera to produce ethanol, acetate, and succinate (Fig. 4c). Under conditions of prolonged anaerobiosis, propionate is preferentially formed as opposed to succinate in anaerobic mitochondria, whereby one additional ATP and one CO_2_ are formed from d-methylmalonyl-CoA via propionyl-CoA carboxylase [25, 37]. We detected expression of a Foraminifera ORF with similarity to propionyl-CoA carboxylase at 28 cmbsf indicating that prolonged anoxic conditions in the sulfidic sediments at 28 cm stimulated production of propionate in Foraminifera mitochondria.

A key intermediate in the anaerobic energy metabolism of most eukaryotes is malate [12, 37]. During anaerobic respiration in many eukaryotes malate is converted to fumarate via the enzyme fumarase running in reverse, and the resulting fumarate then can be used as the terminal electron acceptor [12, 37]. This fumarate reduction is coupled to an anaerobic electron transport chain in which electrons are transferred from NADH to fumarate via a specialized complex I and a mitochondrial membrane associated fumarate reductase [12, 37]. This physiology is typical of anaerobic mitochondria, that are widely distributed amongst eukaryotes including Foraminifera, Bivalvia, Polychaeta, Platyhelminthes, Nematoda, Euglenida, and Ciliophora [12].

**Fig. 6:**
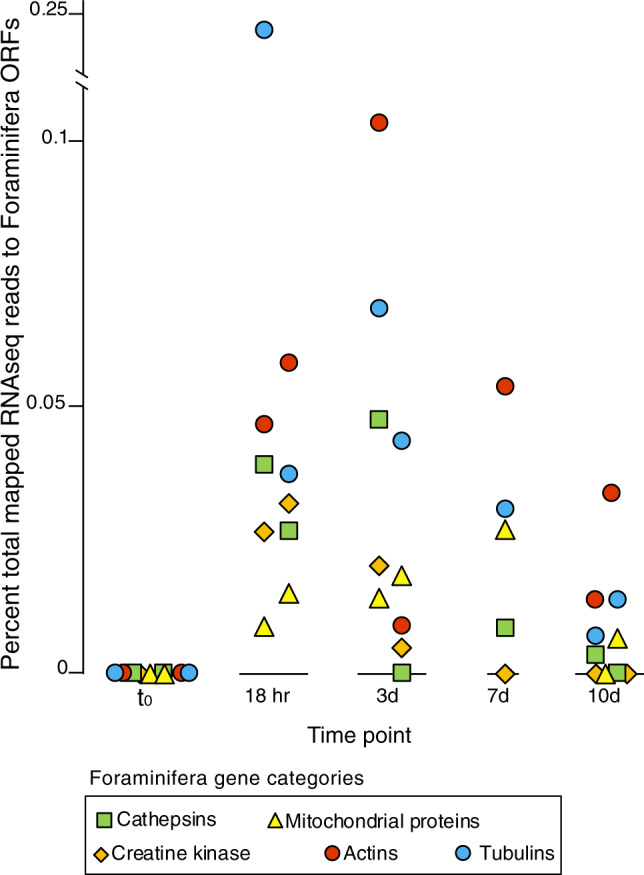
Foraminifera gene categories whose relative expression increased in the presence of anoxia. The five gene categories are shown based on KOG annotations. The category “mitochondrial proteins” are those KOG annotations that have the word “mitochondria”, “mitochondrion”, or “mitochondrial”, in the KOG description. The replicates are shown for each time point are displayed as two individual points, and represent the fractional abundance of all reads mapping to ORFs with a given annotation.

The metatranscriptomes furthermore indicated that under anoxic conditions, Foraminifera utilize creatine kinase and phosphocreatine to maintain cellular energy homeostasis (Fig. 4c). In many eukaryotic cells, creatine kinase acts as a mechanism for maintaining balance between ATP consuming and producing processes [38]. Our data indicate that this also occurs in anaerobic Foraminifera. In human cells, creatine kinase acts as an ATP regenerator, and the phosphocreatine pool is used as a temporal energy buffer to maintain ATP/ADP ratios inside the cell [38]. By acting as an energy shuttle between ATP providing and consuming processes, phosphocreatine acts as a phosphogen to maintain the concentration of the high-energy phosphate pool inside the cell. This facilitates more energetically costly cellular activities under anoxic conditions, such as phagocytosis, by maintaining the spatial “energy circuit” [39]. For example, creatine kinase contributes to the build-up of a large intracellular pool of phosphocreatine that represents an efficient temporal energy buffer and prevents a rapid fall in global ATP concentrations [38]. This likely helps to couple the energy producing and energy consuming processes inside of Foraminifera cells during anaerobic metabolism. An increased utilization of creatine kinase under anoxic conditions is supported by the 10-day incubation, whereby expression of Foraminifera ORFs with highest similarity to creatine kinases, as well as enzymes involving in mitochondrial energy production, increased with the onset of anoxic conditions (Fig. 6).

Biogeochemical studies indicate that foraminiferans are capable of performing denitrification, that is, the conversion of NO_3_^−^ to N_2_ [9]. The enzymes behind the foraminiferal denitrification pathway in the genus *Globobulimina* appear to be acquired relatively early in Foraminifera evolution [10], and it was indicated that the foraminifera themselves, not associated prokaryotes, are performing the denitrification reaction [40, 41]. The sequestration of nitrate by Foraminifera is highly suggestive that the protists themselves, and not associated symbionts, are performing nitrate respiration [40, 41].

Consistent with this prior evidence, we found the genes of the denitrification pathway in Foraminifera to be expressed (Fig. 4), including a putative assimilatory nitrate reductase (Nr). This may function as a sulfite oxidase or dissimilatory nitrate reductase [10, 42]. We interpret the Nr genes to be involved in dissimilatory nitrate reduction with caution and refer to them as “putative nitrate reductases” since it is possible that the Nr genes function solely for nitrate assimilation in Foraminifera [10]. In any case, our data show that these Nr genes are transcribed during anaerobic metabolism in benthic Foraminifera.

The expression of nitrate transporters [10] from Foraminifera at 28 cmbsf (Fig. 4a) seems contradictory to the geochemical conditions, since nitrate and nitrite were both below detection at this depth in the core (Fig. 1). However, this can be explained by the fact that many benthic Foraminifera can store nitrate in vacuoles under anoxic conditions and use the stored nitrate and nitrite as terminal electron acceptors for anaerobic respiration [9, 40, 41]. Thus, the expression of the nitrate transporter genes seen here could be responsible for transporting nitrate out of the vacuole (and regulating the cytosolic concentration of nitrate), and into the mitochondrion, as has been proposed previously for denitrifying Foraminifera based on genome data [10]. The expression of the NirK and Nor genes indicate that the Foraminifera were actively performing two key steps of denitrification—nitrite and nitric oxide reduction (Fig. 4c). Some *Bolivina* and *Stainforthia* and species lack a nitrous oxide reductase and reduce nitrate only to N_2_O [40, 41, 43], and we did not detect any expression of NosZ indicating that the denitrifying *Bolivina* and *Stainforthia* species in our samples were also likely reducing nitrite to nitric oxide, that is then reduced to N_2_O via Nor (Fig. 4c). The lack of expression of the NosZ gene raises the possibility that the denitrifying Foraminifera in Namibian sediments are a source of N_2_O, an important greenhouse gas [44].

The high levels of sulfide in the Namibian sediments should be toxic to eukaryotic life and thus similar to other eukaryotes it would be expected that mechanisms exist to help Foraminifera cope with these conditions. For example, eukaryotic sulfide quinone reductase (SQR) [45] and the sulfide resistant alternative oxidase (AOX) are used by some animals and protozoa to modify their metabolism under anoxic conditions and survive the sulfidic conditions [12]. The KOG database does not contain the SQR, nor AOX genes. After adding all available SQR and AOX genes available in the NCBI Protein database to the KOG database, DIAMOND [46] searches detected one ORF at 12 cmbsf with highest similarity to the Foraminifera *Ammonia* that had significant similarity (amino acid similarity: 46%, alignment length: 259 amino acids, *e*-value: 10^−27^) to AOX from the Oomycete *Pythium aphanidermatum* (Genbank Accession: CAE11918.1). This indicates that some Foraminifera use AOX to perform aerobic fermentation, similar to parasitic trypanosomes, whereby O_2_ is used as the terminal electron acceptor to reoxidize ubiquinol for pyrimidine biosynthesis—as opposed to mitochondrial ATP synthesis [12]. AOX requires O_2_, which we speculate could be available in limited, ephemeral concentrations from the bioirrigating worms that were observed in the core.

The large increase in Foraminifera gene expression upon the onset of anoxic conditions in the incubation (Fig. 5b–d) provides experimental support for the observation of increasing Foraminifera gene expression with increasing depths and sulfidic conditions in the core (Fig. 2c). The higher number of ORFs expressed by Foraminifera after the onset of anoxia (Fig. 5c) indicates that some Foraminifera increased the number of expressed genes, rather than the increase being due to the dying off of other eukaryotes causing an increased relative abundance of Foraminifera transcripts. The increased number of ORFs expressed by Foraminifera could be primarily attributed to those involved in the cytoskeleton, translation, posttranslational modification, and intracellular trafficking (Fig. 5c). This indicates that many Foraminifera were modifying their physiology, increasing translation and protein synthesis in response to anoxic conditions. An increased expression of Foraminifera genes involved in production and modification of the cytoskeleton also suggests that anoxia increased cellular activity [47], rather than causing an increased expression of stress related genes due to unfavorable conditions like the accumulation of hydrogen sulfide. Indeed, no SQR was expressed by Foraminifera during the incubations, which are used by many eukaryotes to cope with sulfidic conditions [12].

Our findings demonstrate that activity of benthic Foraminifera in these sulfidic Namibian sediments is stimulated by anoxic conditions, similar to the findings of a metabolic preference of nitrate over oxygen as an electron acceptor in the Peruvian oxygen minimum zone [11]. The peak stimulation of Foraminifera gene expression after 18 h at the onset of anoxic conditions might indicate the utilization of fumarate, nitrate, and or nitrite by anaerobic denitrifying foraminifera as terminal electron acceptors. This indicates that the *Bolivina* and *Stainforthia* species in the Namibian sediments are anaerobes that prefer anoxic conditions, as this clearly stimulated their activity compared with aerobic conditions.

## Conclusions

The increased gene expression by Foraminifera under sulfidic conditions shows that some Foraminifera apparently not only survive, but are thriving, under anoxic conditions in these anoxic Namibian sediments. Looking at the data, it becomes evident that the anaerobic energy metabolism of these Foraminifera is sufficient to support phagocytosis, CME, and biocalcification under anoxia. The data also confirm that clades of *Stainforthia* and *Bolivina* utilize pathway for denitrification and identified the following pathways of ATP generation including (1) substrate level phosphorylation and fermentation, (2) fumarate reduction, (3) dissimilatory nitrate reduction. Creatine kinases and the dephosphorylation of creatine phosphate appears to play a role in maintaining cellular levels of the high-energy phosphate pool, potentially enabling short lived bursts of energetically demanding activities under anaerobic conditions such as phagocytosis of prey cells. This all indicates that anoxic sediments are a primary habitat of some benthic Foraminifera where they are capable to perform all necessary cellular functions. This anaerobic metabolism is consistent with the evidence for the emergence of Rhizaria in the Precambrian [1, 2] where widespread oxygen depletion was present [48]. This aided the survival of benthic Foraminifera over multiple mass extinctions over the last 500 million years associated with oxygen depletion, thus enabling the utility of their preserved tests as important proxies for paleoclimate and paleoceanography.

## Methods

### Sampling

A 30-cm-long sediment core was obtained from a water depth of 125 m the Namibian continental shelf (18.0 S, 11.3 E) during *F/S* Meteor Expedition M148-2 “EreBUS” on July 10th, 2018. In brief, the core was acquired with a multi corer (diameter 10 cm), which yielded an intact sediment/water interface and the upper 30 cm of sediment. After retrieval, cores were moved immediately to a 4 °C cold room and stored at 4 °C until being sectioned every 2 cm after 20 h. The core was 30 cm long, which was sectioned into 2-cm intervals. Thus, the deepest interval sectioned was between 28 and 30 cm. Sections were transferred immediately into sterile, DNA/RNA free 50 mL falcon tubes and then frozen immediately at −20 °C until DNA and RNA extractions. Pore water geochemistry measurements were performed acquired from the same core, methodology and data have been published elsewhere [31] and the results are reported in this publication in the Fig. 1b.

### Cell counting and enumeration

Between 1 and 4 g of deep-frozen sediment from nine sediment depths were thawed and washed over a 63-micron mesh sieve. The residue was immediately wet-sorted and all test of foraminifera were separated from sediment particles, identified to a genus level following Altenbach and Leiter [26] and enumerated. Representative specimens were photographed using a KEYENCE VHX-6000.

### RNA extraction

RNA was extracted as previously described [31]. In brief, RNA was extracted from 0.5 g of sediment using the FastRNA Pro Soil-Direct Kit (MP Biomedicals) following the manufacturer’s instructions with final elution of templates in 40 µL PCR water (Roche) as described previously [31] with some modifications to maximize RNA yield and reduce DNA contamination. The first modification was that, after the supernatant was removed after first homogenization step, a second homogenization was performed with an additional 500 µL RNA Lysing Buffer. The tubes were centrifuged once again for 5 min at maximum speed, and the supernatant from the second homogenization was combined with that resulting from the first homogenization, continuing with the protocol from the manufacturer. Second, we added glycogen at a concentration of 1 µg/mL during the 30-min isopropanol precipitation in order to maximize recovery of the RNA pellet. To reduce DNA contamination, we extracted all RNA samples in a HEPA-filtered laminar flow hood dedicated only for RNA work (no DNA allowed inside) that also contains dedicated RNA pipettors used exclusively inside the hood with RNA samples. All surfaces were treated with RNAse-Zap prior to extractions and exposed to UV light for 30 min before and after each extraction.

### Metatranscriptomics

Metatranscriptomes were prepared as previously described [31]. In brief, DNAse treatment, synthesis of complementary DNA and library construction were obtained from 10 µL of RNA templates by processing the Trio RNA-Seq kit protocol (NuGEN Technologies). Libraries were quantified on an Agilent 2100 Bioanalyzer System, using the High Sensitivity DNA reagents and DNA chips (Agilent Genomics). The libraries constructed using specific barcodes, pooled at 1 nM, and sequenced in two separate sequencing runs with a paired-end 300 mid output kit on the Illumina MiniSeq. A total of 40 million sequences were obtained after Illumina sequencing, which could be assembled de novo into 41,230 contigs. Quality control, de novo assembly, and ORFs searches were performed as described previously [31], with some minor modifications. In brief, transcripts were trimmed and paired-end reads assembled into contigs using CLC Genomics Workbench 9.5.4 (https://www.qiagenbioinformatics.com/), using a word size of 20, bubble size of 50, and a minimum contig length of 300 nucleotides. Reads were then mapped to the contigs using the following parameters (mismatch penalty = 3, insertion penalty = 3, deletion penalty = 3, minimum alignment length = 50% of read length, minimum percent identity = 95%). Eukaryotic ORFs were detected in contigs using the eukaryotic code for translations and ORF predictions using TransDecoder v5.5.0 [49], whereas bacterial and archaeal ORFs were identified using FragGeneScan version 1.30 [50] with the following arguments -w 1 -t illumina_10.

We did not do an rRNA depletion step, but still recovered mostly mRNA in our libraries. This is partly because the Trio RNA-seq Ovation kit that we used (NuGen technologies) is biased against molecules with secondary structure such as rRNA, and thus preferentially amplifies mRNA. However, BLASTn searches of the remaining reads that did not assemble into contigs confirmed that the unassembled reads were mostly rRNA. Thus, the de novo assembler that we used does not assemble rRNA into contigs, possibly because of its conserved nature. Thus, the vast majority of assembled data is from mRNA because of the preference of the SPIA amplification against molecules like rRNA with secondary structure, and also because of the assembly method used being biased against rRNA.

### Gene identification

A total of 8556 ORFs were found that were then searched for similarity using BLASTp against a database [31] containing predicted proteins from all protist, fungal, bacterial, and archaeal genomes and MAGs in the JGI and NCBI databases using DIAMOND version 0.9.24 [46]. This database, which we refer to as “MetaProt” also contained all ORFs from all of the transcriptomes of microbial eukaryotes from the MMETS project [14] and all of the ORFs from the recently published foraminiferal genome and transcriptome containing the novel denitrification pathway [10]. This custom MetaProt database that we used for this study is available as a single 32 GB amino acid fasta file on the LMU Open Data website (10.5282/ubm/data.183). Cutoff for assigning hits to specific taxa, or to a specific KOG category, were a minimum bit score of 50, minimum amino acid similarity of 30, and an alignment length of 50 residues. We assigned ORFs as being derived from Foraminifera if they had a significant similarity above this threshold to a predicted protein from a previously sequenced Foraminifera transcriptome or genome. Because our database contains predicted proteins from >700 transcriptomes of other microbial eukaryotes, we are confident that this level of stringency is sufficient to make a broad level of taxonomic assignment of ORFs from the metatranscriptomes to Foraminifera in general (as opposed to being actually derived from other protist groups). Normalization of the relative abundance of ORFs (e.g., the relative abundances shown in Figs. 2 and 5) was performed as done previously [31]. Namely, that the number of ORFs assigned per protist group (e.g., Foraminifera) are represented as a fractional percentage, divided by the total number of ORFs with significant similarity (minimum bit score of 50, minimum amino acid similarity of 30, and an alignment length of 50 residues) to a predicted protein from a genome present in the database found with DIAMOND searches.

We normalized expression in this manner, as opposed to more conventional procedures such as RPKM because we found that the RNA-seq kit we used has an amplification step (SPIA amplification, Trio RNA-seq Ovation kit, and NuGen) that biases the relative abundance of reads mapping to contigs when normalized using RPKM. For example, the RPKM value for the same ORF across technical replicates was found to have very large (orders of magnitude) variability in RPKM. In contrast, the total number of unique ORFs (e.g., presence/absence of an expressed ORF) assign to specific groups (e.g., Foraminifera) was highly consistent between technical replicates. We assume that this technical variation in the RNA-seq data is associated with randomized SPIA amplification of different transcripts, and or fluctuations in the number of mRNA molecules in technical replicate tubes due to the highly labile nature of RNA during the extraction and library prep procedure. For this reason, we normalized the relative abundance of ORFs assigned to a specific group based on presence/absence of expressed ORFs which was highly consistent between technical replicates despite the SPIA amplification. If significantly higher numbers of unique ORFs are detected from a particular group compared with other groups it can be attributed to a relatively higher transcriptional activity.

ORFs assigned as Foraminifera were then additionally annotated against the Cluster of KOG database [15], using DIAMOND [46] with the same parameters as above. The lack of metatranscriptomic ORFs having highest similarity to *Bolivina* and *Stainforthia* (Fig. S2) is easily explained by the lack of transcriptome data from representatives of these genera in public databases. Nevertheless, because we cannot be sure from which species each of our metatranscriptome ORF derives, we annotated all of the ORFs having highest similarity to a previously sequenced Foraminifera transcriptome or genome, as being derived from Foraminifera.

Groups of contaminating organisms were identified via the sequencing of 16S rRNA genes from extraction blanks and more than five separate replicates of laboratory dust samples [51]. We used these data to compile a list of all genera present as contaminants in our laboratory dust and extraction blanks, and then removed any ORFs from the metatranscriptomes having significant similarity to predicted proteomes from those same genera in the list. Contaminants were mostly associated with skin and soil associated bacterial genera including *Streptococcus, Acinetobacter, Staphylococcus, Rhizobium, Ralstonia, Pseudomonas*, and *Burkholderia* which are commonly known to be contaminants in molecular reagent kits [52]. All metatranscriptomes had <10% ORFs from contaminating taxa.

### Incubation experiment

Immediately after core retrieval and freezing of the core top samples, 2-g aliquots of sediment from the core top was added to four 20 mL sterile glass vials (for *t*_1_, *t*_2_, *t*_3_, and *t*_4_ timepoints) containing sterile oxygen sensor spots (PreSens Precision Sensing). Oxygen was measured noninvasively using the Fibox (PreSens Precision Sensing) as described previously [53]. The sediment was overlaid with ca. 18 mL of the natural hypoxic bottom water collected in the multicore leaving no air in the headspace, and crimp sealed with gray rubber butyl stoppers. The flasks were incubated on the side and oxygen sensor spots were positioned at the top (to measure oxygen in the overlying seawater) and bottom (to measure oxygen at the base of the sediment) of the flask (see Fig. 5 for a photo of the setup). The flasks were incubated in the dark at 10 °C and taped to the surface of the bench to prevent rolling and mixing of the tube. Each of the four flasks for the timepoints were frozen separately at the respective timepoints *t*_1_ (18 h), *t*_2_ (3 days), *t*_3_ (7 days), and *t*_4_ (10 days) immediately at −20 °C. Because the incubation was setup immediately after core retrieval and freezing the core top samples, the frozen core top samples served as the *t*_0_ samples for the start of the incubation. RNA extractions, metatranscriptomes, and bioinformatic processing was performed as described above.

### Phylogenetics

To identify the likely active foraminifera taxa in the sediments, we searched for foraminiferan 18S rRNA OTUs present within the metatranscriptomes. We performed BLASTn searches (Discontiguous Megablast, *e*-value 1E−10). As query we used a small custom-made database of complete foraminifera sequences based on Pawlowski et al. [54] and Holzmann and Pawlowski [55]. The resulting OTUs were reciprocally blasted against NCBI’s nr database (Discontiguous Megablast, *e*-value 1E−10). The two OTUs with highest similarity to Foraminifera 18S rDNA were further used for sequence extensions using a greedy approach. For this, 10 bp on both ends were trimmed from the putative foraminiferan 18S rRNA OTUs to remove possible erroneous bases due to dropping read quality towards the ends of reads. We only extended the OTU fragment matching the last 1000 bp of the foraminiferan 18S rRNA sequences since this is a commonly used foraminifera barcoding region and allows the comparison with a wide diversity of previously barcoded foraminiferan taxa [56]. We performed 20 iterations of greedy extension in GENEIOUS Prime 2019 [57] by mapping trimmed metatranscriptomics reads with TRIMMOMATIC v.0.38 using the default options [58] to the end-trimmed 18S rDNA OTUs. This extended 5′ and 3′ ends of the 18S rRNA OTUs. Both sequences were manually error corrected based on the mapped reads. We carefully and manually proved that read pairs spanned regions of high sequence similarity with other foraminiferans, i.e., highly conserved stem regions of the 18S rRNA. This approach allowed us to unambiguously extend both OTUs to yield the full 18S rRNA barcoding region. These sequences were blasted against the NCBI nr database and showed strong sequence similarity to the benthic foraminifera genera *Stainforthia* and *Bolivina*. In order to confirm their taxonomic affiliation and to refine their placement, we established two separate alignment that included 30 sequences of the genus *Bolivina* [59] on the one hand, and on the other hand 30 sequences of sister genus *Stainforthia* [60]. The two separate sequence sets were automatically aligned with MAFFT v.7 [61] and a phylogenetic inference was calculated with 1000 nonparametric bootstrapping pseudo replicates based on a BioNJ starting tree using PhyML [62]. The best substitution models were automatically selected using the Smart Model Selection [63] under Akaike Information Criterion and the model GTR + I + G was selected for the *Bolivina* alignment and the model TN93 + G + I was selected for the *Stainforthia* alignment. Both trees were visualized using ITOL and are provided in Fig. 3.

## Supplementary information

Supplemental figure legends

Supplemental Figure S1

Supplemental Table S1

Supplemental Figure S2

## Data Availability

All scripts and code used to produce the analysis have been posted on GitHub (https://github.com/williamorsi/MetaProt-database), and we provide a link to the MetaProt on the LMU Open Data website (10.5282/ubm/data.183) on the GitHub page, as well as instructions within the scripts regarding how to conduct the workflows that we used.
