# Supplementary material for: Anaerobic metabolism of Foraminifera thriving below the seafloor
Source: ISME J. 2020 Jul 8;14(10):2580–94. doi: 10.1038/s41396-020-0708-1 (PMC7490399; doi:10.1038/s41396-020-0708-1)
Supplement: Supplementary file 1 — Supplemental figure legends [file 41396_2020_708_MOESM1_ESM.docx]

**Figure S1: Relative distributions of taxa that Foraminifera-derived ORFs in the metatranscriptome had as top hits after searches with DIAMOND.**

**Figure S2. Schematic representation of trimmed metatranscriptomic reads mapped to the previously sequenced 18S rRNA genes *Bolivina* sp. (A) and *Stainforthia* sp. (B).** The coverage of each fragment is indicated with blue histograms on the top. Each read is shown as black bar. Mapping was performed with GENEIOUS prime as indicated in the methods section.  Note that more reads map to Bolivina, which is consistent with the dominance of cytoplasm bearing tests from Bolivina throughout the core (Fig 1).

**Table S1. Sequencing and assembly statistics.**
