# Supplementary figures and images for: Anaerobic metabolism of Foraminifera thriving below the seafloor
Source: ISME J. 2020 Jul 8;14(10):2580–94. doi: 10.1038/s41396-020-0708-1 (PMC7490399; doi:10.1038/s41396-020-0708-1)

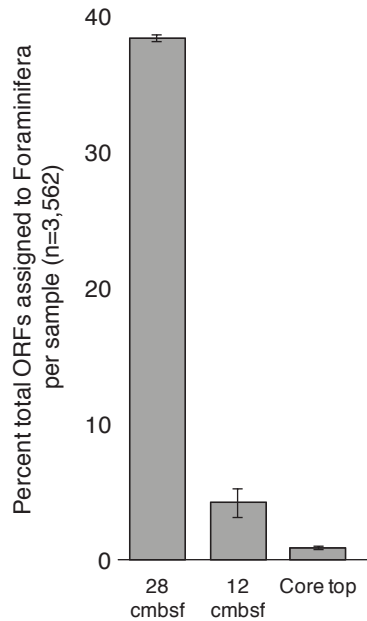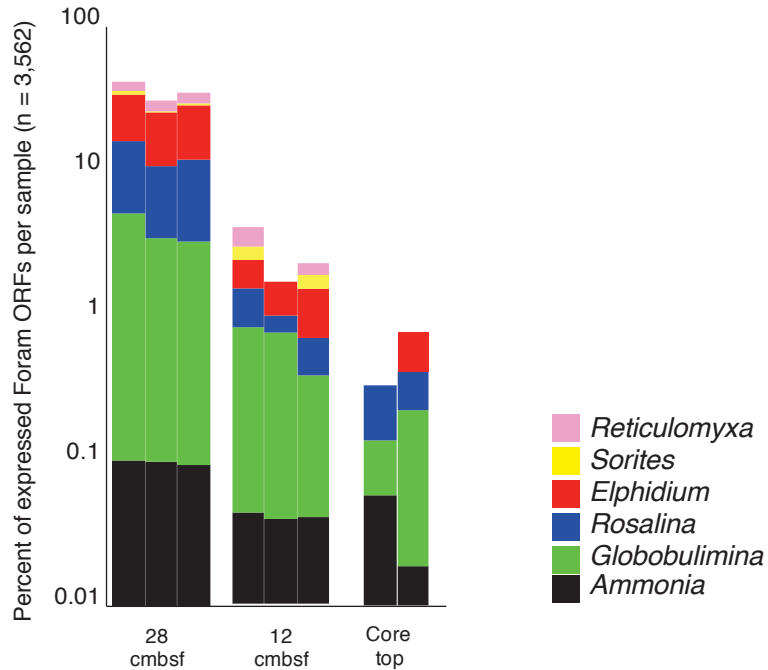

Supplement: Supplementary file 2 — Supplemental Figure S1 [file 41396_2020_708_MOESM2_ESM.pdf]

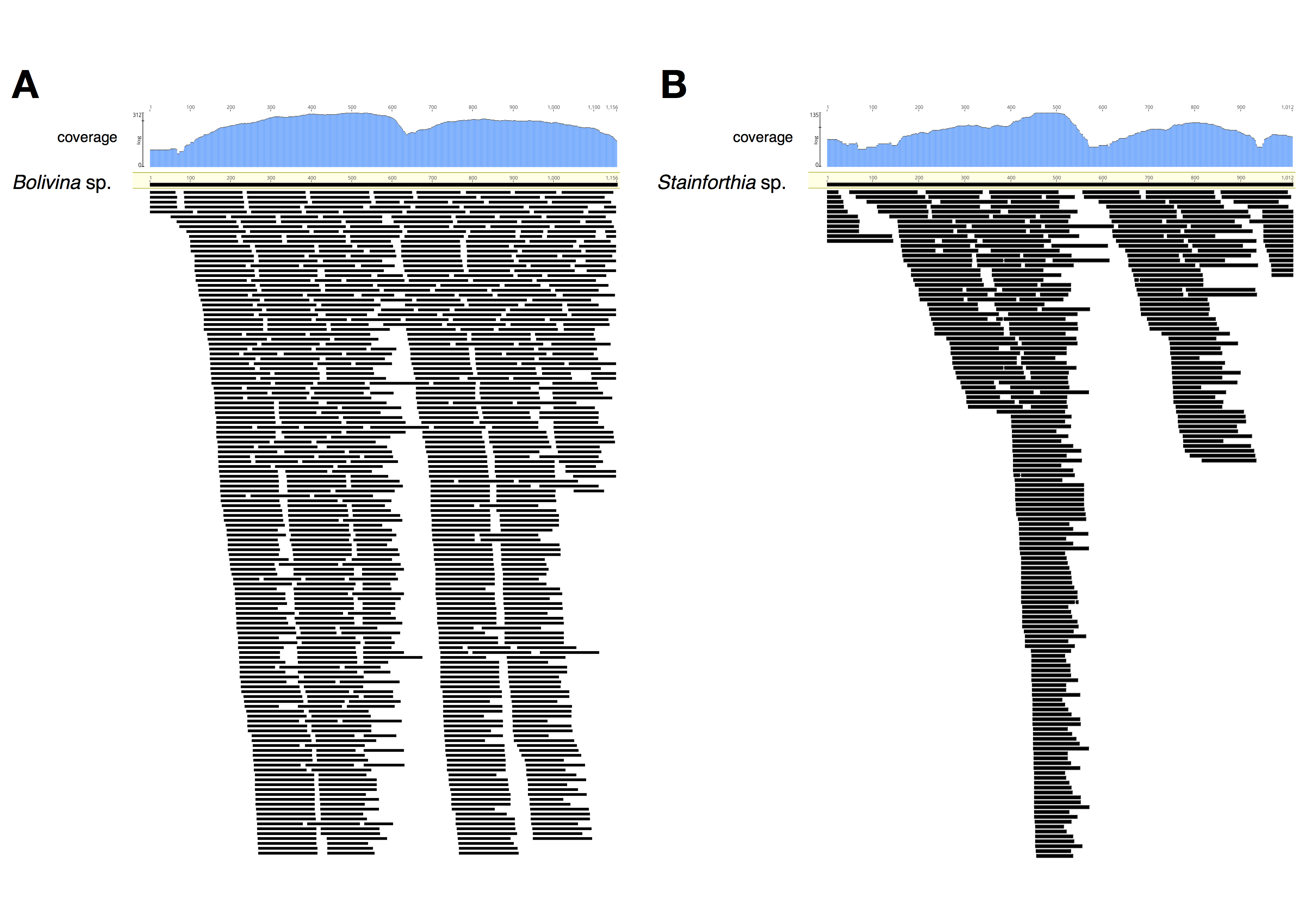

Supplement: Supplementary file 4 — Supplemental Figure S2 [file 41396_2020_708_MOESM4_ESM.png]
