# Supplementary material for: Anaerobic metabolism of Foraminifera thriving below the seafloor
Source: ISME J. 2020 Jul 8;14(10):2580–94. doi: 10.1038/s41396-020-0708-1 (PMC7490399; doi:10.1038/s41396-020-0708-1)
Supplement: Supplementary file 3 — Supplemental Table S1 [file 41396_2020_708_MOESM3_ESM.pdf]

Sample	Replicate	Reads (millions)	# contigs	# reads mapped (millions)	ORFs
core top (frozen, t0)	a	4.7	2,602	3.7	695
	b	11.1	2,927	9.2	687
12 cmbsf (frozen)	a	3.9	4,362	2.7	539
	b	2.2	2,726	1.3	1,075
	c	3.5	5,888	2.1	1,113
28 cmbsf (frozen)	a	3.9	7,429	2.4	995
	b	5.8	9,636	4.2	1,993
	c	4.1	5,660	2.9	1,459
incubation 18 hours	a	2.1	3,854	0.83	996
	b	2.2	3,811	0.73	947
incubation 3 days	a	3.0	7,167	1.2	1636
	b	2.7	3,860	1.3	913
incubation 7 days	a	2.9	4,222	1.3	904
incubation 10 days	a	2.7	2,463	1.0	553
	b	2.3	2,279	1.2	543
